# Errata

**DOI:** 10.36660/abc.20210278

**Published:** 2021-04-08

**Edição de Outubro de 2019, vol. 113 (4), págs. 787-891**

Na “Atualização da Diretriz de Prevenção Cardiovascular da Sociedade Brasileira de Cardiologia – 2019”, com número de DOI: https://doi.org/10.5935/abc.20190204, publicado no periódico Arquivos Brasileiros de Cardiologia, 113(4):787-891, na página 862, corrigir o item “Não-HDL-colesterol” da tabela 11.3, “> 145” para “< 145”, nas colunas 1 e 2; na página 863, corrigir o item “Não-HDL-colesterol”, de Lípides, da tabela 11.6, “> 145” para “< 145”, nas colunas 1 e 2.

**Edição de Janeiro de 2021, vol. 116 (1), págs. 160-212**

No “Posicionamento sobre o Consumo de Gorduras e Saúde Cardiovascular – 2021”, com número de DOI: https://doi.org/10.36660/abc.20201340, publicado no periódico Arquivos Brasileiros de Cardiologia, 116(1):160-212, na página 160, corrigir o nome da autora Lis Mie Misuzawa Beda para: Lis Mie Masuzawa Beda.

