# Supplementary material for: Errata
Source: Arq Bras Cardiol. 2021 Apr 8;116(4):855. [Article in Portuguese] doi: 10.36660/abc.20210278 (PMC8121396; doi:10.36660/abc.20210278)
Supplement: Translation

# Translation: Erratum

**October 2019 Issue, vol. 113 (4), pages 787-891**

In the “Updated Cardiovascular Prevention Guideline of the Brazilian Society of Cardiology – 2019”, with DOI: https://doi.org/10.5935/abc.20190204, published in the journal Arquivos Brasileiros de Cardiologia, on page 862, correct item “Non-HDL-cholesterol” in table 11.3, “> 145” to “<145”, in columns 1 and 2; on page 863, correct the item “Non-HDL-cholesterol”, in Lipids, from table 11.6, “> 145” to “< 145”, in columns 1 and 2.

**January 2021 Issue, vol. 116 (1), pages 160-212**

In the “Position Statement on Fat Consumption and Cardiovascular Health – 2020”, with DOI: https://doi.org/10.36660/abc.20201340, published in the journal Arquivos Brasileiros de Cardiologia, 116(1):160-212, on page 160, correct author name Lis Mie Misuzawa Beda to: Lis Mie Masuzawa Beda.
